# A binary-based approach for detecting irregularly shaped clusters

**DOI:** 10.1186/1476-072X-12-25

**Published:** 2013-05-06

**Authors:** Tai-Chi Wang, Ching-Syang Jack Yue

**Affiliations:** 1 Department of Statistics, National Chengchi University, NO.64, Sec.2, ZhiNan Rd., Wenshan District, Taipei City 11605, Taipei, Taiwan, ROC

**Keywords:** Spatial cluster detection method, Choynowski’s test, Binomial approximate method, Permutation test, Spatial scan statistic

## Abstract

**Background:**

There are many applications for spatial cluster detection and more detection methods have been proposed in recent years. Most cluster detection methods are efficient in detecting circular (or circular-like) clusters, but the methods which can detect irregular-shaped clusters usually require a lot of computing time.

**Methods:**

We propose a new spatial detection algorithm for lattice data. The proposed method can be separated into two stages: the first stage determines the significant cells with unusual occurrences (i.e., individual clustering) by applying the Choynowski’s test, and the second stage determines if there are clusters based on the information of the first stage by a binomial approximate method. We first use computer simulation to evaluate the performance of the proposed method and compare it with the scan statistics. Furthermore, we take the Taiwan Cancer data in 2000 to illustrate the detection results of the scan statistics and the proposed method.

**Results:**

The simulation results support using the proposed method when the population sizes are large and the study regions are irregular. However, in general, the scan statistics still have better power in detecting clusters, especially when the population sizes are not large. For the analysis of cancer data, the scan statistics tend to spot more clusters, and the clusters’ shapes are close to circular (or elliptic). On the other hand, the proposed methods only find one cluster and cannot detect small-sized clusters.

**Conclusions:**

In brief, the proposed methods can detect both circular and non-circular clusters well when the significant cells are correctly detected by the Choynowski’s method. In addition, the binomial-based method can handle the problem of multiple testing and save the computing time. On the other hand, both the circular and elliptical scan statistics have good power in detecting clusters, but tend to detect more clusters and have lower accuracy in detecting non-circular clusters.

## Background

Spatial patterns of diseases are of interest to both epidemiologists and the general public because they often link the incidence of disease with suspected agents or environment factors. The intent of epidemiologists, then, is usually to investigate whether the clusters occur in specific areas at certain times. A local cluster is defined as the area with unusual higher or lower intensity caused by some unobserved effects [1]. The definition of the local cluster, further, are categorized into global clustering and cluster detection methods [2].

The spatial cluster detection methods are concerned with the locations of the detected spatial clusters. Initially, the geographical analysis machine (GAM) [3] was proposed to determine the spatial clusters via circular windows. Based on this idea, the population size and the number of cases were used to determine the significance of clusters [1,4]. Most methods encounter the multiple testing problem because their algorithms construct many elective regions to be tested. The Kulldorff’s spatial scan statistic constructs a series of circular scan windows to detect the most likely cluster and uses a Monte Carlo approach to evaluate the significance of the located cluster to avoid the multiple testing problem [5].

In these methods, disease clusters are usually assumed to be circular, and thus most spatial cluster detection methods use circular windows or expand circularly to detect clusters. This assumption, however, does not always reflect the actual pattern of diseases which do not always radiate out in a circular form. Clusters may appear along a river because water is a vehicle for the transmission of some infectious diseases; for example, the mosquito larval habitat mainly located around the river and was a major cause of West Nile Virus [6]. Besides, the clusters may be affected by the wind direction; for example, the vibrio cholera dissemination was related to this [7]. The circular windows look especially awkward in Taiwan, since the Taiwan island and most of its counties are not rectangular or alike-circular. For example, the clusters of epidemics in Taiwan were likely to take a sinuous or long shape, rather than a circular one [8,9]. Some reports also mentioned that the cancer incidence rate and the cancer mortality rate in Taiwan were generally higher in the mountain and downstream river areas [10]. The male bladder cancer mortality rates on average showed that higher mortality rates (i.e., hot spots) appear along the downstream rivers, which is an irregularly long-shaped cluster in Taiwan’s west plain from 1992 to 2001.

Several modifications of cluster detection have been proposed to deal with irregularly shaped clusters. The upper-level set scan statistic [11] collected the connected components of all upper level sets to be the suspected clusters. The flexible scan statistic (FleXScan) [12] also proposed a connection algorithm to detect irregular clusters. A minimum spanning tree algorithm [13] was developed to construct the possibly irregular-shaped clusters and then to test them. The spatial scan statistic (SaTScan) with elliptic version [14], and the trajectory method [15] are also well-known methods for detecting clusters of irregular shape. Many studies, meanwhile, compared the power and accuracy of cluster detection methods [16-20]. These modified methods generally obtain better results in detecting irregularly shaped clusters. However, most of these methods also adopt the Monte Carlo testing procedure, but this procedure of the irregular detection methods will cost more computing time than that of circular methods. This seems inefficient in practice. Thus, we propose a two-stage approach for identifying irregular clusters without spending too much computing time.

Note that the proposed detection method is designed to deal with non-circular clusters for aggregate data. Unlike the previous modifications, however, the proposed method transforms the data into a binary form and computes the significance via an approximate binomial distribution. This computing procedure can save computing time without using a Monte Carlo procedure. The developed two-stage approach can reduce the suspected clusters and computation time for determining the locations of clusters. In addition to the theoretical development, we compare the proposed method with Kulldorff’s circular and elliptical scan statistics (SaTScan), whose software is presented on their web-sites and is open to access, and explore whether the proposed method offers better performance in detecting irregularly shaped clusters.

## Methods

The goal of this study is to determine if there exists local clusters, that is, regions with higher relative risks or disease incidence rates in the study area. In particular, the focus is on developing a method which can identify irregularly shaped clusters. Also, the proposed method should be suitable to deal with aggregate data or lattice data, because most data in many countries are collected at the county level or the township level and rarely appear in the format of an individual level.

It should be noted that the neighborhood structure is one of the key features of the lattice data, and that it usually contains important information of spatial data. The proposed method will take the neighborhood information into account for identifying clusters. Basically, we use the adjacent neighborhood information to connect cells. Based on the number of connected neighbors, a binomial-based method can be embedded in the proposed method, and it can significantly reduce the computing time. We shall first define the notations to facilitate the description of the proposed method.

### Notations

Suppose the study area, *S*, is divided into *k* mutually exclusive cells, such as counties, townships, or census tracts. Let *S*_*i*_ be the *i*^*th*^ location, and *Z*(*S*_*i*_) be the interested quantity, such as the disease incidence rate in lattice data. Besides, if one attempts to study the disease incidence, the observed number of cases and the number of at-risk individuals (or at-risk population size), defined as *T*_*i*_ and *N*_*i*_, respectively, must be taken into account. Meanwhile, let the total number of cases be *T*_+_ and total number of individuals at risk be *N*_+_. Under the null hypothesis of no clustering, the number of observed cases *T*_*i*_ in location *S*_*i*_ is assumed to be independent of those in other locations and to follow a Poisson distribution. Also, suppose *E*(*T*_*i*_)=*λN*_*i*_, *i*=1,2,…,*k*, where *λ* is the overall disease incidence rate or the overall mortality rate, which can be estimated as the overall mean of the observations, *T*_+_/*N*_+_.

### The binomial approximate method

The proposed method can be separated into two stages: the first stage determines the significant cells with unusual occurrences (i.e., individual clustering), and the second stage determines if there are clusters based on the information supplied by the first stage. Because most existing methods evaluate many elective regions (i.e., suspected clusters), they take lots of computing time to identify clusters and may not be empirically efficient. The two-stage design of the proposed method can reduce the number of elective clusters to be tested via approximating a binomial-based probability of the connected regions.

This method does not require information regarding cluster shapes or locations. Basically, it can be used to detect single and multiple clusters. Also, since the proposed approach is a two-stage design, we need to define two significance levels (namely, *α*_1_ and *α*_2_) to determine the clusters. Later, we shall give a more detailed discussion about these two parameters.

### Stage 1. Clustering test of individual cell (Choynowski’s test)

The first stage of the proposed method is to check whether there are cells containing unusually large numbers of occurrences. This idea is adopted from the Choynowski’s test [21] to test whether there are clustering patterns for each lattice cell. The steps of testing are as follows: 

1. Estimate the overall disease incidence rate or mortality rate *λ*, by $\hat{\lambda }=T_{+}/N_{+}$.

2. Estimate the expected number of disease cases in cell *S*_*i*_, *e*_*i*_, by ${ê}_{i}=\hat{\lambda }N_{i}$.

3. Suppose *Z*(*S*_*i*_) denoted the number of disease cases in cell *S*_*i*_ to be a random variable. Under the null hypothesis of no clustering, *Z*(*S*_*i*_) is assumed to follow the Poisson distribution with the mean ${ê}_{i}$ defined above. We can then calculate the p-value of cell *S*_*i*_ of the first stage, i.e.,

(1) $$p_{i}^{\left(1\right)}=\text{Pr}(Z(S_{i})\geq z(S_{i}))=\underset{Z(S_{i})\geq z(S_{i})}{\sum }\frac{\exp(-{ê}_{i}){ê}_{i}^{Z(S_{i})}}{Z(S_{i})!}.$$

4. Record the cells with unusually high occurrences, i.e., with p-value smaller than a predetermined significance level *α*_1_, to be the significant cells.

### Stage 2. Cluster detection

In this stage, the significant cells identified in the first stage are treated as the centers of suspected clusters and then we determine if these suspected clusters are the real clusters by evaluating the “connected probabilities”, which will be defined later. Although we are interested in methods which can detect arbitrarily shaped clusters, we also understand that circular clusters is a popular choice in practice. Thus, we shall evaluate if the proposed method is efficient in detecting circular clusters.

In addition, the neighborhood information (the default setting is adjacent neighbors) is an important element in the proposed algorithm. Suppose the significant cells identified in the first stage are treated as “black” cells. Then, the second stage is used to decide whether these “black” cells can connect into real clusters. For each “black” cell *i*, we check if its neighbors are also “black”, and record the number of “black” neighbors as *B*_*i*1_. The number of “black” neighbors can be treated as a random variable following a binomial distribution, *Bin*(*n*=*ℵ*_*i*_,*p*=*α*_1_), in which the parameter ‘ *ℵ*_*i*_’ is the number of neighbors, and the success probability ‘ *α*_1_’ is equivalent to the significance level of the first stage. Then, the probability of observing the number of “black” neighbors of cell *S*_*i*_ with the size *b*_*i*1_ under the null hypothesis can be expressed as

(2) Pr(Bi1≥bi1|H0)=1−Pr(Bi1≤(bi1−1)|H0)=1−∑Bi1=0bi1−1ℵiBi1α1Bi1(1−α1)ℵi−Bi1.

This is the first step to evaluate the probability of forming a possible cluster from *S*_*i*_ to its significant neighbors.

In the second step, the same algorithm as the first step is applied to evaluate if the cluster with center *S*_*i*_ can expand to a wider region. To avoid the cells to be counted twice or more, we only compute the new involved cells, that is, the cells connected with the new significant neighbors which are expanded from *S*_*i*_ but not connected with other clustered cells. Thus, we can define a new binomial random variable *B*_*i*2_, and then keep on computing the same procedures till no significant neighbors can be included as the clustered cells. Under the null hypothesis that all cells are independent, let *M*_*i*_ as the steps of forming the connecting region from *S*_*i*_, and the approximate probability of observing such a *M*_*i*_ step pattern is

(3) $$p_{i}^{\left(2\right)}=\overset{m_{i}}{\underset{j=1}{\prod }}\text{Pr}(B_{\text{ij}}\geq b_{\text{ij}}|H_{0}),$$

where *m*_*i*_ is the connected steps from *S*_*i*_. This probability is defined as the “connected probability” of *S*_*i*_.

It should be noted that the number of diseases in a cell follows a discrete distribution, and thus the p-value of the critical point is not necessarily equal to *α*_1_, unless a randomized test is adopted. Also, every cell has a different critical point, and it would be inefficient to calculate all critical points. Instead, we use the equation (3) to approximate the true probability, although the approximate probability may be larger. Of course, the randomized test can be used to confine the equation (3) such that its significance level is exactly equal to *α*_1_.

After the first stage, all cells lost their original information (e.g., population size) and are transformed into binary data (black and white). Still, we can determine clusters based on the binary data by introducing a “expanding probability.” The expanding probability, similar to the type II error, is computed for diagnosing if there is any possible expansion, and it is the probability that no additional neighbored cells of the cluster can be included in the cluster given that they are parts of clusters (the alternative hypothesis). This probability can also be computed via a binomial distribution,

(4) $$\begin{array}{rl} \;\text{Pr}(\text{no neighbors are significant}|H_{1})= & \underset{S_{j}\in \delta_{i}\setminus {\mathbb{C}}_{i}}{\prod }\text{Pr}(Z(S_{j})\\ & <C_{j}^{\left(0\right)}|H_{1}), \end{array}$$

where *δ*_*i*_ is the neighbor set of the cluster with the center *S*_*i*_, ${\mathbb{C}}_{i}$ is the clustered cells of the cluster with the center *S*_*i*_, *S*_*j*_ is an element of $\delta_{i}\setminus {\mathbb{C}}_{i}$, where ‘ ∖’ is the set subtracted operator, and $C_{j}^{\left(0\right)}$ is its critical point under the null hypothesis *H*_0_. If the expanding probability is lower than a predetermined value *β*, the algorithm stops and reports the connected probability of the suspected cluster with the center *S*_*i*_. Otherwise, the suspected cluster will include the neighbor of the suspected cluster with the lowest p-value, and we call this neighbor as a “junction” point. Then, we add the events and population of the “junction” point into its neighbors, and treat them as new elective clustered cells. The process of expanding the clusters continues until reaching the stopping criterion.

The binomial approximate method also suffers the multiple testing problem. However, it can be adjusted by the Bonferroni correction because all the suspected clusters are independent under the null hypothesis. Thus, a suspected cluster will be treated as real one when the connected probability of it is smaller than *α*_2_/*B*, where *B* is the number of total suspected clusters. It should be noted that a single significant cell can not be treated as a suspected cluster since it is impossible to compute the connected probability.

We shall also give some comments about the proposed approach. First, it is possible that more than two centers form the same cluster, but the connected probabilities of them are different. We would choose the one with the highest connected probability. Second,a suspected cluster with more neighbors will have a lower probability to expand when there are no significant neighbors. Finally, the binomial approximation would become less reliable when there are more significant cells, since the independent assumption between cells is less likely to be true. We should introduce a permutation test as a possible alternative to the binomial approximation.

### Permutation test

If there are a lot of significant cells identified in the first stage, the preceding approximation would be not feasible in practice. Then, we can use permutation test to find the potential clusters. The idea is to check whether the suspected cluster with the maximum connected “black” number is significant or not. Although we only consider the case of one cluster, the permutation test can easily be modified to detect multiple clusters. The testing p-value is obtained by the following procedures.

Suppose there are *b* significant cells out of *n* cells from the first stage, and the maximum elective cluster consists of *M* connected cells. The permutation test in the following procedures is used to check if the number of maximum connected significant region, *M*, is unusually large. 

1. Randomly permute *b* significant cells out of the total *n* cells for *G* times (999 or 9,999). That is, suppose the permutation data are (*X*_1_,*X*_2_,…,*X*_*k*_). Each *X*_*i*_ is randomly assigned a binary value (0 or 1) and is confined by $\sum_{i=1}^{k}X_{i}=b$. For each simulation run, compute and record the maximum number of connected cells as the largest cluster.

2. Suppose the maximum number of connected cells in the *g*th permutation is *L*_*g*_. Then, the permutation p-value for testing under there are no clusters is obtained as

$$\text{Pr}(L_{b}\geq M)=\frac{\#{\left\{L_{g}\geq M\right\}}_{g=1}^{G}+1}{G+1}$$

If the p-value is smaller than or equal to the pre-decided significance level *α*_2_, then we conclude that the suspected cluster with M connected cells is indeed a cluster.

### Example

**Figure 1:**
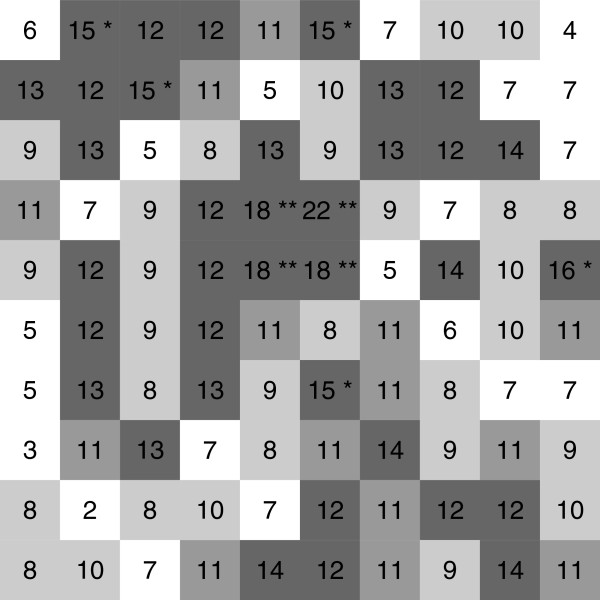
**The image plot for the counts of disease.** The image plot shows the counts of disease in each location. The ** represents the p-value is smaller than 0.05, and the * represents that is smaller than 0.1 under the null hypothesis *λ*=10.

Let the study region be a squared grid with 10 × 10 squares, and each cell follows a binomial distribution with *n*=10,000 and *p*=0.001 (approximate to a Poisson distribution with *λ*=10) except the specified 2 × 2 cluster located in the center of the study area with a higher expectation *λ*=20 (*p*=0.002) (Figure 1). In this example, the equal population case is used to simply illustrate the computing procedure.

Following the procedures of stage 1, we first compute the estimated overall disease incidence rate, $\hat{\lambda }=103/10000=0.0103$. After estimating all $\hat{e_{i}}$, we can identify the significant cells via a predetermined *α*_1_=0.1. As shown in Figure 1, there are 9 significant cells (the cells of value 15 are just on the significant boundary at *α*=0.1, so we include them as significant cells), the “black” cells, under the significant level *α*_1_=0.1. We see that the values {18, 22, 18, 18} in the central area are identified as a significant cluster.

According to the procedures of stage 2, it needs two steps to form the full connection. The probability of the first step for expanding from one center to it’s significant neighbors is 0.0523 (a center with 4 neighbors and 2 of them are significant at *α*=0.1). Similarly, the probability of the second step is 0.4095 (a clustered region with 5 new neighbors and 1 of them is significant at *α*=0.1). Thus, the “connected probability” is 0.0214 (there is only one suspected cluster and the Bonferroni correction is not required) and the expanding probability is very small (<0.0001). Other than these values, no other significant cells are connected, and thus we only have to determine if the region with connected “**” cells is a cluster.

On the other hand, we execute the permutation test for 999 runs, that is, randomly permute the 9 significant cells out of 100 cells. In this permutation result, there is only one run in which the cluster size is larger or equal to 4. Thus, the p-value via the permutation test is 0.039. Obviously, these two probabilities are not the same.

Similar to the binomial approximation, we found that the single significant cell can not be a cluster using the permutation test. Nonetheless, the proposed approach still benefits from imposing fewer constraints. For example, most detection methods require certain assumptions, such as the size of cases, the range of distance, and the shape of cluster. However, the proposed method relies heavily on the testing results of first stage. This is the reason why we proposed the expanding probability, which makes the binomial approximate method more flexible and can be used to detect non-circular clusters. In the following subsection, we use computer simulation to evaluate the proposed method and compare it with the scan statistics.

### Evaluate the proposed methods and the SaTScan

In this part, two computer simulation studies are conducted to evaluate the proposed methods: one with cells of equal population in a regular grid area and the other with actual population (i.e., unequal population) in Taiwan island. The detailed settings of these simulations are mentioned later.

Usually, both the type-I error and the power are used to evaluate a test. However, since the power provides little information regarding the locations and sizes of the clusters, we will use other measurements to evaluate the accuracy of cluster detection. We shall first define the terms of true positive, false positive, true negative, and false negative. True positive (TP) cells are the true clustered cells which are correctly detected as clusters; false positive (FP) cells are the non-clustered cells which are incorrectly detected as clusters; true negative (TN) cells are the non-clustered cells which are not identified as clusters; false negative (FN) cells are the true clustered cells which are not identified as clusters. The sensitivity, defined as TP/(TP+FN), is used to measure the proportion of identified clustered cells among all true clustered cells. In addition, we suggest using the error rate, which is defined as

(5) $$\text{ER}=\frac{\text{FP}+\text{FN}}{\text{TP}+\text{FP}+\text{FN}},$$

to evaluate the false detection rate.

#### Simulation 1: equal population case with 20 by 20 regular grid

**Figure 2:**
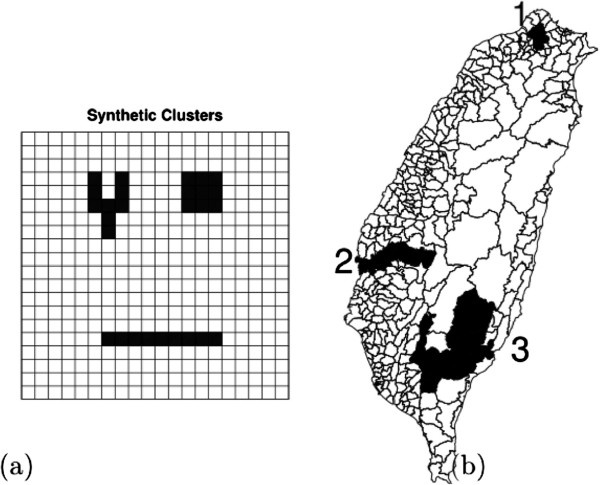
**Synthetic clusters in the two study areas.** (**a**) Regular 20 by 20 grid and (**b**) Taiwan Map. The darker areas are the locations of the synthetic clusters.

The first simulation study addresses a grid area with 20 by 20 squared cells in which each cell has identical at-risk population (10,000). There are two scenarios: one is a no-cluster case for checking the type-I error and the other is an one-cluster case for evaluating the power and the detection accuracy of the proposed methods. For the no-cluster case, we assume that each cell has the same incidence rate of disease. For the one-cluster case, the cells within the cluster have a higher disease incidence rate of disease than those outside the cluster. In addition, the cluster is either circular-shaped, long-shaped, or Y-shaped, and every cluster is of size 9, as shown in the left panel of Figure 2.

### No-cluster model

The goal in the no-cluster case is to check if the proposed method can achieve the predetermined significance level. Both the binomial approximate method and the permutation test will be evaluated. Let two significance levels *α*_1_ and *α*_2_ in stage 1 and stage 2 be 0.01, 0.05, or 0.10. The stopping criterion of the expanding probability for the binomial approximation is suggested to be conservative and is 0.001 in this study.

For the no-cluster case, we assume the disease incidence rate is 0.001 and the population size is 10,000 in each cell. The results of 1,000 simulation runs for a no-cluster case are shown in Table 1. For the permutation method, the results indicate that the four combinations of *α*_1_ and *α*_2_, i.e., *α*_1_=0.05 or 0.10 vs. *α*_2_=0.05 or 0.10, provide good approximate to the predetermined significance level, *α*_2_, but no combinations of *α*_1_=0.01 or combinations of *α*_2_=0.01 give satisfactory results. On the other hand, the binomial method produces close approximations to the predetermined significance level at *α*_1_=0.05 or 0.10 vs. *α*_2_=0.05.

**Table 1:** Type I error of proposed methods

	Binomial^*^			**Permutation**		
	***α***_**2**_			***α***_**2**_		
***α***_**1**_	** 0.1**	** 0.05**	** 0.01**	** 0.1**	** 0.05**	** 0.01**
0.1	0.119	0.073	0.025	0.108	0.059	0.009
0.05	0.098	0.061	0.024	0.113	0.055	0.014
0.01	0.035	0.021	0.009	0.073	0.039	0.007

* The expanding probability is 0.001 and the Bonferroni criterion is used to modify the multiple testing problem.

In general, for both the binomial approximate method and the permutation test, we recommend using *α*_1_=0.05 and *α*_2_=0.05. Nevertheless, if it is difficult to detect clusters, for example, when the relative risk (RR: the disease incidence ratio of cluster cells to non-cluster cells) is low (fewer significant cells), we recommend using the combination of *α*_1_=0.1 and *α*_2_=0.05 to accumulate enough significant cells for the testing. Note that the setting *α*_1_=0.05 vs. *α*_2_=0.05 will be used as the default setting in the rest of this study.

### One-cluster model

According to the previous results, the proposed methods achieve the predetermined type I error. To further check the performance of cluster detection by the proposed methods, the cluster set in the 20 by 20 grid area consists of 9 cells, and it can be of the circular shape (3 by 3), long shape (1 by 9), or Y-shaped. The left panel of Figure 2 shows shapes and their corresponding locations in the 20 by 20 grid area. Each cell in this area is with equally background intensity rate 0.001 and equal population size 10,000. In addition, the relative risk (RR) of the clusters ranges from 1.5 to 3 steps by 0.5.

**Figure 3:**
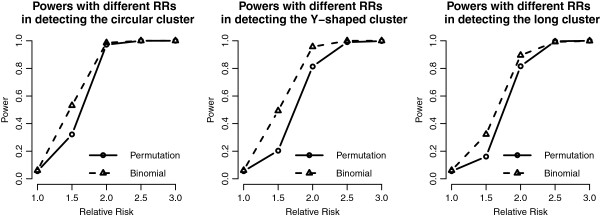
**Power curves of the proposed methods in the 20 by 20 grid area.** Power curves of the proposed methods with different RRs and cluster shapes in the 20 by 20 grid area. Note that the value represents the type I error when RR = 1.

Figure 3 shows the powers of the binomial approximate method and the permutation test, and generally both methods have higher powers as the RR becomes higher. Because the binomial approximate method has an adjustment of junction cell, it has higher power than the permutation method and thus higher type I error as well (Table 1). Both methods have lower powers on detecting long cluster and higher powers on detecting the circular one. The power of both methods is almost 1 when the RR is at least 2.

**Figure 4:**
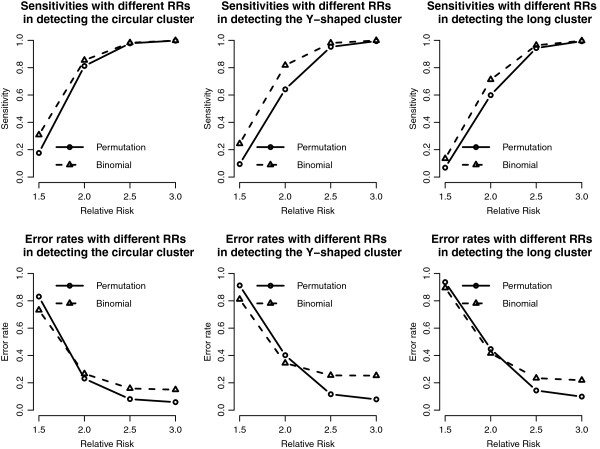
**Sensitivity and error rate curves of the proposed methods in the 20 by 20 grid area.** Sensitivity and error rate curves of the proposed methods with different RRs and cluster shapes in the 20 by 20 grid area.

Figure 4 shows the sensitivity and the error rates for the proposed methods. For the sensitivity, both the binomial approximation and permutation test perform well when the RR is big (e.g., 2.5 and 3), but not as good when the RR is low (1.5 and 2.0). On average, the binomial approximate method has the better sensitivity than the permutation test. On the contrary, the error rates show the opposite results. The permutation test has lower error rates especially for the case of larger RR, due to the fact that the binomial approximate method might include extra junction (not clustered) cells, and might result in a wider expansion of the detected cluster (or higher false positive probability).

#### Simulation 2: Unequal population case in Taiwan island

For the sake of practical considerations, we also consider the one-cluster case with the actual population’s distribution according to the townships in Taiwan. Like what we did in “simulation 1”, we intend to add three different levels of the population for the three different clustered types, low, median, and large. However, it would be too lengthy to discuss all combinations. In addition, we found the simulation results are very similar to the regular case for some combinations. Thus, we present only the cases which can show the differences between the proposed method and SaTScan. In specific, we choose the three clusters whose regions resemble Taiwan’s township structure to be the synthetic clusters with unequal population.

We take the observed HIV prevalence rate of adults (15–49 years old), which was estimated as 0.0003 in Taiwan 2003 [22], to be the background disease incidence rate, and the adult proportion is approximately 60% of total population.

There are 350 townships in Taiwan, close to 400 cells in regular grid data, but the characteristics of each township (e.g., shape, population size, and neighborhood structure) are dramatically varied. Like in many countries, the population sizes are very different in rural and urban counties. In Taiwan, the maximal and minimal population sizes are 1,745 and 523,850, respectively. In addition, because Taiwan is an island country, the shape and the number of neighboring townships of each township vary a lot. The smallest township is only 5.9 square kilometers, while the largest is 1641.8 square kilometers. We want to explore if the detection results would be influenced by the geographic attributes of Taiwan townships.

We will only show the results of one-cluster case, since the efficiency of cluster detection is of interest. The simulated clusters can be seen in the right panel of Figure 2. The first cluster is set to be circular and its population size is twice as large as the average population size (the average size of ages 15-49 in 350 Taiwan townships is about 37,588.). The second cluster is set to be long and its population size (about 21,677) is approximately equal to the median of all townships. The third cluster is set to be Y-shaped and has the lowest population size (just 6,498).

**Figure 5:**
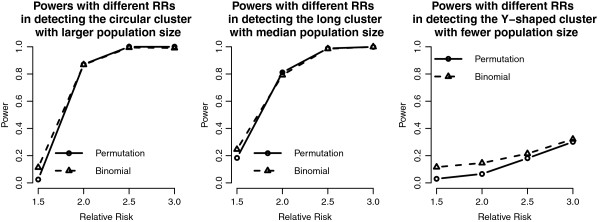
**Power curves of the proposed methods in Taiwan geographical region.** Power curves of the proposed methods with different RRs and cluster shapes in Taiwan geographical region.

**Figure 6:**
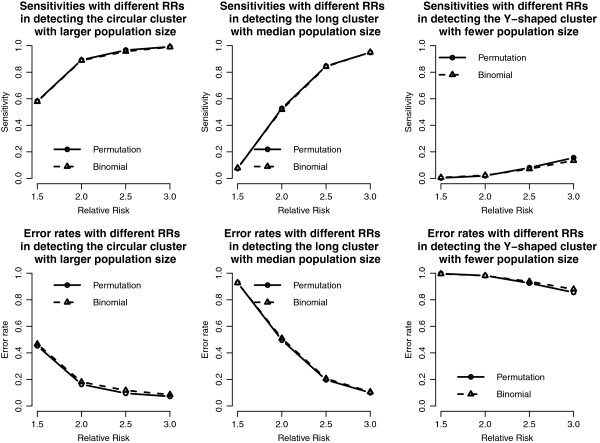
**Sensitivity and error rate curves of the proposed methods in Taiwan geographical region.** Sensitivity and error rate curves of the proposed methods with different RRs and cluster shapes in Taiwan geographical region.

We use the same RR setting as those in the 20 by 20 grid case, except that the background intensity rate is chosen as the average prevalence rate of HIV (about 0.0003). Compared to the case of 20 by 20 grid, the simulation results in the case of imposing real populations show that population sizes play an important role in cluster detection. If the population size is 6,498 (Cluster 3 with Y-shaped), neither the power (Figure 5), sensitivity, nor error rate (Figure 6) show satisfactory results even when the RR reaches 3. Because the expected numbers of cases just approximate to 2 in the clustered cells under the null hypothesis, the expected numbers of observed cases are just 6 even when the RR is 3. These numbers might not be large enough to be identified as significant in the probability regime. Other than the small population case, the proposed methods have good powers and small error rates in the other two cases, similar to those in the 20 by 20 grid case.

#### Power comparisons with the scan statistics

The scan statistic (SaTScan) [23] is one of the popular methods to detect spatial clusters. It is a kind of likelihood ratio test and is especially powerful in detecting circular clusters. It sets multiple scanning windows, constructed from centers of cells with gradually increasing radius, and tests if the interested variable in the selected window is significantly different from that outside the window. The test statistic can be expressed as

(6) $$\lambda =\frac{\sup_{Z\in Z,p>q}L(Z,p,q)}{\sup_{p=q}L(Z,p,q)}=\frac{L(\hat{Z})}{L_{0}},$$

where *Z* is the selected window, *p* is the intensity rate in the region *Z*, and *q* is the intensity rate outside *Z*. The testing procedure is based on the Monte Carlo method. For each simulation run, the disease cases are randomly distributed into the study region according to the population size. Other than the original circular window, an elliptical method was also proposed to construct elective windows [24]. In this study, both the original (i.e., circular) and elliptical windows of SaTScan are considered. The SaTScan software can be downloaded from http://www.satscan.org.

We shall use the simulation to compare the proposed methods with the SaTScan. The focus is on the performance of cluster detection. Again, we apply the same simulation settings on the 20 by 20 grid and the Taiwan synthetic data.

**Figure 7:**
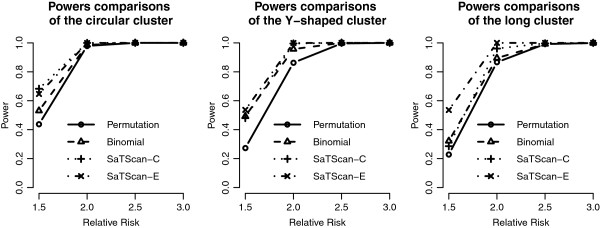
**Power comparisons in the 20 by 20 grid area.** Power comparisons of the proposed methods and the SaTScan methods with different RRs and cluster shapes in the 20 by 20 grid area.

**Figure 8:**
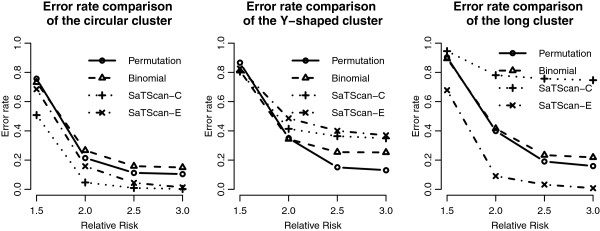
**Sensitivity and error rate comparisons in the 20 by 20 grid area.** Sensitivity and error rate comparisons of the proposed methods and the SaTScan methods with different RRs and cluster shapes in the 20 by 20 grid area.

We add the detection results of the scan statistics into Figures 3, 4, 5 and 6 to see the differences among them. First, by observing the power comparisons in the case of 20 by 20 grid area (Figure 7), the SaTScan methods have better power than the proposed methods, and the elliptical SaTScan has the best power among them, especially in detecting the long cluster. Figure 8 clearly shows the differences among these methods. Although the powers of the elliptical and circular SaTScan are better than the proposed methods, the proposed methods have the better sensitivity and lower error rates in the case of detecting the Y-shaped cluster. The elliptical and circular SaTScan also have obvious differences; the elliptical SaTScan is especially good in detecting the long cluster, and the circular SaTScan is the best to detect the circular cluster.

**Figure 9:**
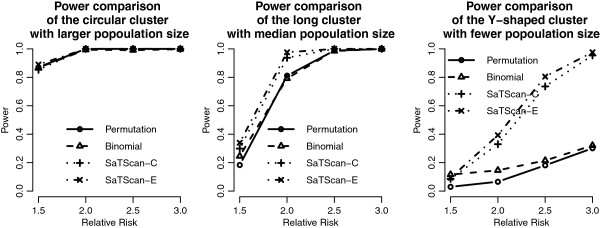
**Power comparisons in Taiwan geographical region.** Power comparisons of the proposed methods and the SaTScan methods with different RRs and cluster shapes in Taiwan geographical region.

**Figure 10:**
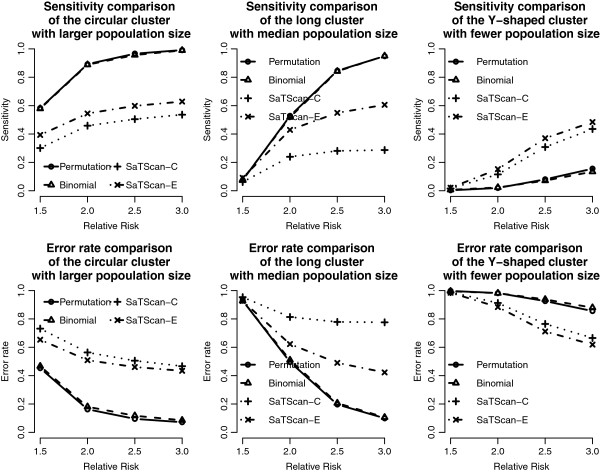
**Sensitivity and error rate comparisons in Taiwan geographical region.** Sensitivity and error rate comparisons of the proposed methods and the SaTScan methods with different RRs and cluster shapes in Taiwan geographical region.

Similar to Figure 6, we can also evaluate the detection performance of the SaTScan methods for different population sizes. As shown in Figure 9, these four methods have almost the same power in detecting the circular cluster, but the proposed methods have significant drops when the population size is fewer (Cluster 3). On the other hand, the SaTScan methods are more consistent even the population size is very small. In Figure 10, the sensitivity and the error rates are also used to evaluate the performances among these methods. The proposed methods have the better error rates and sensitivity in detecting clusters except for the fewer population case. This result is interesting since the circular and elliptical SaTScan are designed to detect circular and long clusters.

The simulation study shows diverse results and no single detection method can outperform other methods. Nevertheless, we would give the following suggestions. If the population sizes are large and the study regions are irregular, the proposed methods is a better choice than the SaTScan. In addition, if computation time is a major concern, the binomial method is preferred because it does not require the Monte Carlo procedure. If there is little information about the shapes of clusters or the population of them, the SaTScan methods are recommended due to their good testing powers.

### Application: Taiwan cancer data

In addition to computer simulation, we also use real data to evaluate the proposed methods. In particular, the Taiwan cancer data (death records) in year 2000 are used, since cancer is the top cause in Taiwan for more than 25 years. Since the cancer related mortality rates increase as people become older, we shall focus on the population of the elderly (ages 65 and over). Also, we shall separately explore whether there are clusters for the elderly groups of male, female, and both-sex combined. The cancer mortality data were from the Ministry of Interior (MOI), Taiwan government. The mortality records are maintained by the MOI and are available to the academic institutes (including universities and research organizations), after removing personal information.

**Figure 11:**
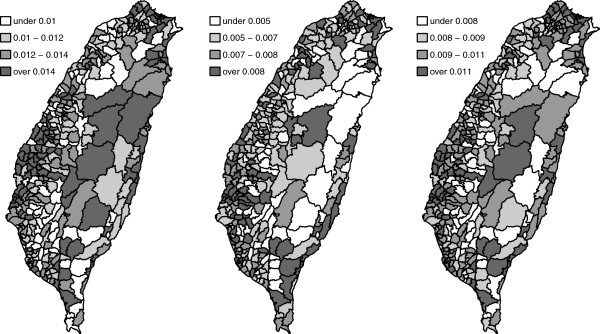
**The mortality rates of Taiwan elderly in 2000.** From left to right: male, female, and both for the Taiwan elderly.

We first consider the cancer mortality rates for each township (Figure 11). A darker color represents a higher mortality rate. Apparently, the northern coast and the middle western areas have higher mortality rates, no matter for the male, female, and both-sex.

**Figure 12:**
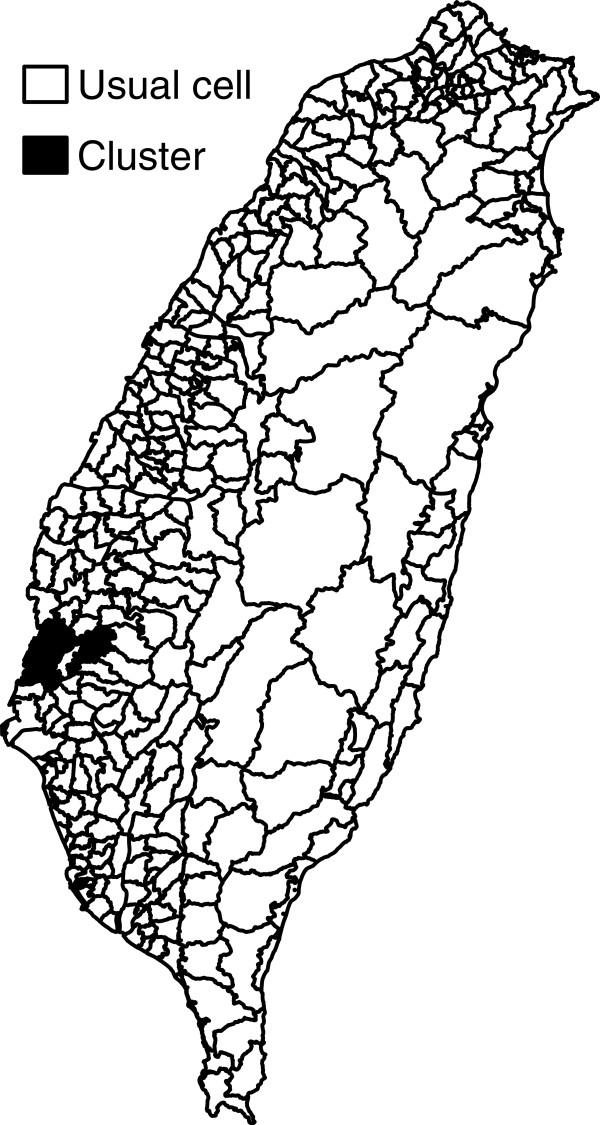
**The cluster detected by the proposed method.** The cluster with higher caner mortality rate detected by the proposed method for Taiwan female elderly in 2000.

Other than the female elderly, the proposed methods do not find any significant clusters. For the female elderly case (Figure 12), two proposed methods detect identical cluster, at the same location and with the same size. Using the proposed binomial approximate method, the p-value of the cluster is 0.0002 with 5 multiple comparisons, comparing to the p-value 0.002 of the permutation method using 999 permutations. The cluster contains 6 cells and its shape is not close to circular. The female cancer mortality rate in the clustered region is 0.0116 (the relative risk is approximately 1.706) and average female population of each cell is 2,274. These clustered cells locate in Tainan County and Chiayi County, two agricultural counties. Also, the proportions of the elderly are higher in the clustered area, which is 0.1370, comparing to 0.0845 for Taiwan’s average in 2000. It seems that inside the cluster, the population structure and cancer mortality rate of the elderly are quite different from those outside the cluster.

**Figure 13:**
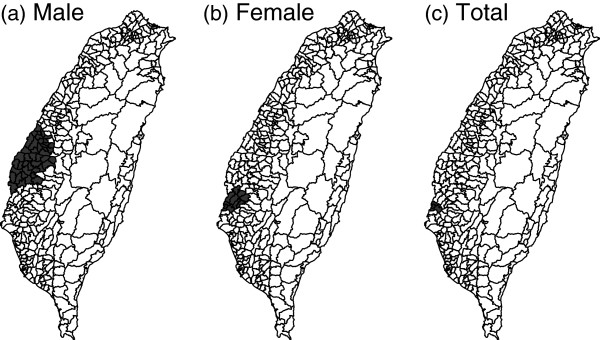
**The first clusters detected by the circular SaTScan.** The first clusters with higher caner mortality rate detected by the circular SaTScan method for Taiwan elderly in 2000.

**Figure 14:**
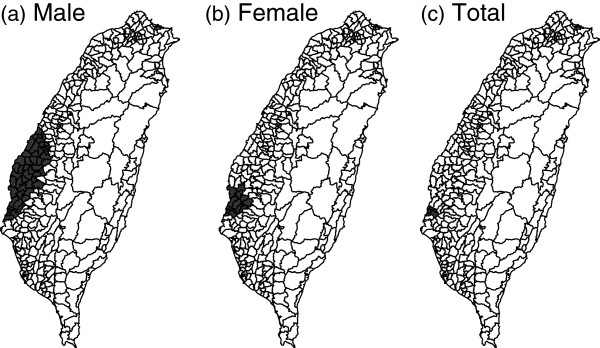
**The first clusters detected by the elliptical SaTScan.** The first clusters with higher caner mortality rate detected by the elliptical SaTScan method for Taiwan elderly in 2000.

For comparison, we also apply the SaTScan methods to these data. Unlike the proposed method, the SaTScan methods detect more than one cluster, but we show only the first significant clusters for comparison. On the other hand, the circular SaTScan and elliptical SaTScan usually detect different clusters, which can be seen from Figures 13 and 14. Basically, clusters detected by these two methods have overlaps, but the circular SaTScan tends to detect round clusters while the elliptical SaTScan detects long clusters.

From the analysis of cancer data, we can see more differences between the proposed methods and SaTScan. As expected, the SaTScan is more powerful in detecting clusters. Thus, it tends to spot more clusters and is also more likely to commit error in finding false positive cells. Also, the SaTScan uses scanning windows to detect clusters and their shapes would be close to circular (or elliptic). On the other hand, the proposed methods rely on the connecting probability to spot clusters and therefore cannot detect small-sized clusters. For example, the cluster spotted by the SaTScan in both-sex elderly group consists only 2 cells, even its relative risk is fairly large (around 2).

## Discussion

Although the proposed method performs better at detecting irregularly shaped clusters in our simulations, it still has some drawbacks. For example, the accuracy of detection heavily depends on the significant cells determined in the first stage. If the RR of the potential cluster is not very large or it has a small population size, the proposed method might misjudge the true clusters. For example, if a center cell of a long cluster is misjudged as insignificant, the true cluster will be broken into two pieces. Therefore, if a cell is significant, then its neighbor cells must be treated with extra care. This is the reason why we set a flexible junction point. Another possible modification is to consider reducing the threshold of the significance level for a cell in the first stage. However, this can result in a higher type I error and too many significant cells from the first stage might distort the binomial approximation.

Another limitation of the proposed method is that a cluster is determined by its size (i.e., the number of connected significant cells). A set of a larger number of connected cells is more likely to be treated as a cluster, and a cluster of small size (e.g., one or two cells) is barely detectible. This problem can be modified by considering the weighted case (i.e., the population connected) instead of counting the number of connected significant cells. This modification can easily be adopted in the permutation-based method, but it is more complicated to embed the modification in the binomial approximate method.

Note that the permutation method is currently used to detect if there is one cluster. This can be modified to detect two or more clusters by removing the first cluster and its adjacent cells, then repeating another permutation test. In this manner, the study region will therefore be changed, and this change would increase the difficulty of applying the permutation test. Nonetheless, such modification for detecting multiple clusters seems to be fine conceptually, and it has been checked by means of simulation in the case of two clusters. We will continue to explore whether the proposed approach performs well in detecting more than two clusters.

In addition, the binomial approximate method can be expanded to a generalized linear model (GLM). After fitting the model, we can obtain the residuals and determine which cells are different from others (outliers). Then, we can adopt the same procedures to compute the connecting probability and identify the clusters. However, if the data contain clusters, a regular GLM is likely to give biased estimations depending on the characteristics of these clusters. In other words, it is not easy to separate the effects of GLM and clusters, and the cluster detection would become more complicated [25].

## Conclusion

In this study, we proposed an approach which can detect clusters with shape not restricting to circular (or elliptic). The proposed approach is a two-stage method, and is designed for data at an aggregate level, such as township data. It uses a traditional Poisson test (Choynowski’s test) to determine if a cell has a clustering pattern (i.e., contains too many disease cases) or is an outlier, and then uses a binomial approximate method to compute a p-value to check if there are clusters. In addition, we also develop a permutation-based method to compute the exact p-value of suspected clusters. Unlike most cluster detection methods where the scanning windows are applied, using the two-stage method has the advantage of computational efficiency.

We use computer simulation and empirical data to evaluate the proposed methods, and compare them with the frequently used method, the SaTScan. Overall, the SaTScan methods detect more and larger clusters than the proposed methods. The elliptical SaTScan has the best power and also has lowest error rates in detecting long and circular clusters of the regular grid data. On the other hand, we found that the proposed methods have the best error rates and sensitivity in detecting irregularly shaped clusters when the population sizes are large. In general, the elliptical SaTScan has the best performance in cluster detection, and this explains why the SaTScan is very popular. Still, if the clusters tend to be of irregular shape, we recommend checking the detection results of proposed methods with those of SaTScan methods.

We know that there are other detection methods for irregular shaped clusters. In fact, we did compare the proposed method with the FleXScan (freeware http://www.niph.go.jp/soshiki/gijutsu/download/flexscan/), but the FleXScan takes a lot of computing time in the simulation study. In our experience, the FleXScan can detect irregularly shaped clusters well when the cluster areas are small, such as 4 or 5 cells. If the clusters widely expand, the detecting parameter would be large, resulting in more computing time. Instead, we include the elliptical SaTScan, in addition to the original circular SaTScan, to avoid unfair judgment.

## Competing interests

Both authors declare that they have no competing interests.

## Authors’ contributions

T-CW carried out the Binomial method, performed the statistical analysis of the simulation and empirical studies, and drafted the manuscript. C-SJY proposed the main ideas and the permutation test, participated in its design and coordination, and helped to draft the manuscript. Both authors read and approved the final manuscript.
